# Inhibitory activity of medicinal mushroom *Ganoderma lucidum* on colorectal cancer by attenuating inflammation

**DOI:** 10.1093/pcmedi/pbab023

**Published:** 2021-08-28

**Authors:** Mandy M Liu, Tiantian Liu, Steven Yeung, Zhijun Wang, Bradley Andresen, Cyrus Parsa, Robert Orlando, Bingsen Zhou, Wei Wu, Xia Li, Yilong Zhang, Charles Wang, Ying Huang

**Affiliations:** Department of Pharmaceutical Sciences, College of Pharmacy, Western University of Health Sciences, Pomona, CA 91766, USA; Center for Genomics & Department of Basic Sciences, School of Medicine, Loma Linda University, Loma Linda, CA 92350, USA; Department of Pharmaceutical Sciences, College of Pharmacy, Western University of Health Sciences, Pomona, CA 91766, USA; Department of Pharmaceutical Sciences, College of Pharmacy, Marshall B. Ketchum University, Fullerton, CA 92831, USA; Department of Pharmaceutical Sciences, College of Pharmacy, Western University of Health Sciences, Pomona, CA 91766, USA; College of Osteopathic Medicine of the Pacific, Western University of Health Sciences, Pomona, CA 91766, USA; Department of Pathology, Beverly Hospital, Montebello, California, CA 90640, USA; College of Osteopathic Medicine of the Pacific, Western University of Health Sciences, Pomona, CA 91766, USA; Department of Pathology, Beverly Hospital, Montebello, California, CA 90640, USA; Beijing Tong Ren Tang Chinese Medicine Co., Ltd., New Territories, Hong Kong 999077, China; Beijing Tong Ren Tang Chinese Medicine Co., Ltd., New Territories, Hong Kong 999077, China; Beijing Tong Ren Tang Chinese Medicine Co., Ltd., New Territories, Hong Kong 999077, China; Beijing Tong Ren Tang Chinese Medicine Co., Ltd., New Territories, Hong Kong 999077, China; Center for Genomics & Department of Basic Sciences, School of Medicine, Loma Linda University, Loma Linda, CA 92350, USA; Department of Pharmaceutical Sciences, College of Pharmacy, Western University of Health Sciences, Pomona, CA 91766, USA

**Keywords:** *Ganoderma lucidum*, colorectal carcinoma, RNA-seq, NF-κB, inflammation

## Abstract

The medicinal mushroom *Ganoderma lucidum* (GL, Reishi or Lingzhi) exhibits an inhibitory effect on cancers. However, the underlying mechanism of the antitumor activity of GL is not fully understood. In this study, we characterized the gene networks regulated by a commercial product of GL containing a mixture of spores and fruiting bodies namely “GLSF”, in colorectal carcinoma. We found that *in vitro* co-administration of GLSF extract at non-toxic concentrations significantly potentiated growth inhibition and apoptosis induced by paclitaxel in CT26 and HCT-15 cells. GLSF inhibited NF-κB promoter activity in HEK-293 cells but did not affect the function of P-glycoprotein in K562/DOX cells. Furthermore, we found that when mice were fed a modified diet containing GLSF for 1 month prior to the CT26 tumor cell inoculation, GLSF alone or combined with Nab-paclitaxel markedly suppressed tumor growth and induced apoptosis. RNA-seq analysis of tumor tissues derived from GLSF-treated mice identified 53 differentially expressed genes compared to normal tissues. Many of the GLSF-down-regulated genes were involved in NF-κB-regulated inflammation pathways, such as IL-1β, IL-11 and Cox-2. Pathway enrichment analysis suggested that several inflammatory pathways involving leukocyte migration and adhesion were most affected by the treatment. Upstream analysis predicted activation of multiple tumor suppressors such as α-catenin and TP53 and inhibition of critical inflammatory mediators. “Cancer” was the major significantly inhibited biological effect of GLSF treatment. These results demonstrate that GLSF can improve the therapeutic outcome for colorectal cancer through a mechanism involving suppression of NF-κB-regulated inflammation and carcinogenesis.

## Introduction

Colorectal cancer is the third most common type of cancer and also the third most common cause of cancer-related death in both men and women in the United States.^1^ Currently, surgery and chemotherapy are the main treatment options for colorectal cancer, depending on the stage, grade, and tumor location at diagnosis. Chemotherapy is used for patients with metastatic colorectal cancer as primary therapy, and for patients with early-stage colorectal cancer before or after surgery as adjuvant therapy. However, chemoresistance is a common obstacle that occurs in almost all cases of colorectal cancer. In addition, because chemotherapy non-selectively affects all cells that are active in the cell growth cycle, it causes toxicity in normal organs such as the bone marrow, gastrointestinal tract, and hair follicles. Therefore, the common clinical practice is to use a combination of several anticancer agents with different mechanisms of actions or toxicity profiles to overcome drug resistance and reduce toxic reactions.

Globally, it has become more and more popular for cancer patients to use complementary and alternative medicine derived from natural sources during or after the course of conventional anticancer therapies. A nationwide survey conducted in Japan revealed a prevalence of herbal use in 44.6% of cancer patients and in 25.5% of non-cancer patients with benign tumors, and the most frequently used herbal products were mushrooms.^2^ One of the most well-known medicinal mushrooms is *Ganoderma lucidum* (GL), also commonly named as Reishi or Lingzhi, which has been used for centuries in East Asia to treat a variety of disorders, including inflammation and cancer, without any obvious toxicity.^3,4^ GL and related products are referred to as ‘The Mushroom of Immortality’ because of beneficial effects on health and longevity. The spores of GL are the reproductive cells of the fungus, which are ejected from the cap after the fruiting bodies become mature. The major bioactive components identified in GL spores and fruiting bodies are polysaccharides, which are known to stimulate the immune system, as well as triterpenes, which may directly inhibit proliferating cancer cells.^5^

Anticancer activity has been investigated using different parts of the GL mushroom, the spores, fruiting bodies or mycelia (the early harvest), in human and preclinical animal models. The evidence related to colorectal cancer can be summarized into three types of studies. First, GL has shown efficacy in certain preclinical models of colon cancer prevention, that is prophylaxis. In several reports, the water extracts of the mycelia culture media of GL prevented chemical carcinogen-induced colorectal carcinogenesis in mice or rats.^6–8^ The triterpene extract prevented colitis-associated colon carcinogenesis in mice.^9^ Feeding water extract of GL powder to rats with a high-fat diet reduced fecal secondary bile acids and intestinal bacteria known to be related to colon carcinogenesis.^10^ GL polysaccharide consumption reduced 30% of mortality, specific bacteria and expression of cancer-related genes in mice with colon cancer induced by chemical carcinogens.^11^ Second, GL has shown some clinical evidence of preventing development of colon cancer or modulating some biomarkers related to immunomodulation or carcinogenesis. For example, the same mycelia extract mentioned above suppressed the number and size of precancerous colorectal adenomas in human subjects who took the mycelia extract at 1.5 g/day for 12 months.^12^ In patients with advanced colorectal cancer, taking oral GL at 5.4 g/day for 12 weeks induced some changes in immune-related parameters such as IL-1 and TNF-α, although the effects were not statistically significant.^13^ Third, GL has shown some antitumor efficacy in a few preclinical studies of human colon cancer cell lines *in vitro*. For example, the effect of enzymatically hydrolyzed GL polysaccharide has been examined in human colon cancer cell lines, indicating inhibition of apoptosis via up-regulation of BCL-2 associated X protein (Bax) and down-regulation of COX-2.^14^ However, besides reports of the prophylactic efficacy for GL, few studies have reported the therapeutic effects of GL products as a single therapy or combined with conventional chemotherapies for already diagnosed colorectal cancer both *in vitro* and *in vivo*. Therefore, it is largely unknown whether colorectal cancer patients could benefit from treatment with GL products.

The present study aimed to determine the effect of GL on colorectal cancer *in vitro* and *in vivo*. A commercial GL product containing a mixture of spores and fruiting bodies, namely “GLSF”, was used. We firstly evaluated whether the GLSF extracts prepared in several methods including the use of artificial gastrointestinal juice (to simulate *in vivo* digestion) have any direct anticancer effects on cancer cells in culture. As colorectal cancers are commonly resistant to taxanes, we next evaluated whether artificial gastrointestinal juice digested GLSF has any chemosensitizing effect in combination with paclitaxel *in vitro*. Furthermore, we evaluated whether a GLSF modified diet was able to inhibit tumor growth in a mouse model bearing murine colorectal carcinoma CT26, which is one of the most extensively used syngeneic mouse tumor models.^15^ Tumor samples dissected from GLSF-treated and control diet-treated tumor-bearing mice were analyzed by RNA sequencing (RNA-seq). Potential mechanisms of the anticancer and chemosensitization effect of GLSF in colorectal carcinoma were further explored through bioinformatics analysis.

## Materials and methods

### Compounds and reagents

Paclitaxel (T7402) purchased from Sigma Aldrich (St Louis, MO) was used *in vitro*. Abraxane® for injectable suspension (Celgene, Summit, NJ) was used *in vivo*. A single batch of a commercial product manufactured by Beijing Tong Ren Tang Chinese Medicine Co. (Hong Kong, China), named as GLSF, contains a mixture of the spores and fruiting bodies of GL at a 30:8 ratio, was used throughout the present study.

### Preparation of GLSF extracts and quantification of the major components

As there is no standardized preparation approach for GL and related products, several reagents were used to extract GLSF including ethanol, methanol, hot water, as well as artificial gastric and intestinal (GI) juice. For the ethanol/methanol extract, 0.5 g of GLSF was extracted with 12.5 ml solvent in a round bottom flask attached to a reflux condenser at 80 °C for 2 hours. The mixture was centrifuged at 4500 g for 10 minutes. The supernatant was collected, and the pellet was extracted by repeating the steps above. The supernatant collected was combined and filtered using Whatman filter paper. The solvent was removed using a rotary evaporator.

For the hot water extract, 10 g of GLSF was extracted with 200 ml of nanopure water at 85 °C for 15 minutes under stirring. The mixture was then centrifuged for 15 minutes at 4500 g. The supernatant was filtered using Whatman filter paper and filtrate lyophilized. The powder obtained was weighed and stored at −20 °C before analysis.

The extracts of GLSF were prepared in the artificial gastrointestinal juice, which was prepared according to reported methods.^16,17^ In brief, 5 g of GLSF powder was mixed in 50 ml of artificial gastric juice at 37 °C with shaking for 1 hour, then 50 ml of artificial intestinal juice was added and incubated at 37 °C with shaking for an additional 5 hours. The mixture was centrifuged at room temperature for 15 minutes at 4500 g, and the supernatant was collected. The extract was then neutralized to pH 7.0 using 0.2 M NaOH, filtered using Whatman filter paper, lyophilized, and stored at −20 °C.

The major active components were determined using a validated HPLC-DAD method (unpublished), which was able to quantify 13 major components in GLSF including ganoderenic Acid C, ganoderic Acid C2, ganoderic Acid G, ganoderic Acid B, ganoderenic Acid B, ganoderic Acid A, ganoderic Acid H, ganoderenic Acid D, ganoderic Acid D, ganoderic Acid F, ganoderic Acid DM, ganoderol A, and ergosterol. The individual contents in the extracts were quantified and used as a fingerprint for GL products. All the extractions and fingerprint analysis were conducted in triplicate.

### Cell lines and cell culture

CT26, HCT-15, HT-29, and HEK-293 cells were purchased from ATCC (Manassas, VA). K562/DOX cell line (a gift from J.P. Marie, INSERM, E9912, University of Paris, France) was obtained by *in vitro* passaging of K562 in progressively increasing doses of daunorubicin.^18^ These cells were maintained in RPMI-1640 or DMEM supplemented with 10% fetal bovine serum and 1% penicillin-streptomycin. Cells were incubated at 37 °C with 5% CO_2_/95% air.

### SRB cell proliferation assay

Plates (96-well) were seeded with 3000 cells per well and the cells were allowed to attach overnight. Cells were treated with drugs for 72 hours and incubated at 37 °C in 5% CO_2_/95% air. Cell viability was determined using a Sigma sulforhodamine B (SRB) assay according to the manufacturer's protocol.

### Apoptosis analysis

Flow cytometry was used to assess apoptosis by FITC-labeled annexin-V (Sigma) and propidium iodide, respectively, as previously described.^19^ Briefly, 1 × 10^5^ cells were seeded per well in six-well tissue culture plates for 24 hours. The cells were then incubated with drugs for 72 hours. Cells were collected and washed twice with cold PBS. Approximately 5 × 10^5^ cells were mixed with binding buffer with or without Annexin V or PI. Fluorescence was detected in fluorescence channels FL1 and FL3. Data acquisition and analysis were performed using Accuri C6 Flow Cytometer (BD Biosciences, Franklin Lakes, NJ). Analysis was based on acquisition of data from 10 000 cells. Early apoptotic cells are Annexin-V positive and PI-negative, whereas late apoptotic cells are both Annexin-V and PI-positive.

### Intracellular daunorubicin (DNR) accumulation assay

The capacity of a compound to inhibit P-glycoprotein-mediated efflux from K562/DOX cells, was measured by flow cytometry as described previously.^20^ Briefly, the cell pellet was resuspended in culture media at a concentration of 6 000 000 cells/ml. Aliquots (50 μl) of the cell suspension were transferred to tubes containing 1.95 ml of media in the presence or absence of test drugs in media and 5 μM of DNR for 50 minutes at 37 °C. After centrifugation and removal of supernatant, cold PBS was added to each tube, and cell suspension was transferred to FACS tubes, which were placed on ice until analysis. Flow cytometry was performed with an Accuri C6 Flow Cytometer. PSC833 (10 μM) served as a positive control.

### Luciferase reporter gene assay

Dual luciferase assay for NF-κB has been described in previous work.^21^ In brief, HEK-293 cells were transfected with pRL-TK-Luc *Renilla* luciferase (Promega, Madison, WI) and pGL4.22-NF-κB (Promega) plasmids at a ratio of 1:30 using FuGENE HD Transfection Reagent (Roche Applied Science, Indianapolis, IN). Twenty-four hours later the cells were exposed to TNF-α (10 ng/ml) and incubated in fresh growth medium containing drugs for an additional 5 hours. Cell lysates were analyzed by the dual luciferase reporter gene assay (Promega) with *Renilla* luciferase serving as a normalization factor.

### Rodent diet preparation

For *in vivo* studies, 1.25% of GLSF powder was incorporated into mouse diets for oral administration. The modified animal diet was ordered through Newco Distributors, Inc (Rancho Cucamonga, CA). The formulation included 98.45% Laboratory Rodent Diet #5001, 1.25% GLSF, and 0.3% color dye. The calculation of the drug was based on an average daily intake of 4 g per mouse. Food consumption for each group was monitored during the study and it was confirmed that the dose of GLSF was ~2.0 g/kg.

### Animal experiment

All animal studies were carried out in strict accordance with the recommendations in the Guide for the Care and Use of Laboratory Animals of the National Institutes of Health and approved by the Western University of Health Sciences Institutional Animal Care and Use Committees. A pilot study was conducted using five male and six female BALB/c mice. CT26 cells (3 × 10^6^) in 100 µl serum free media were injected subcutaneously into the right and left flanks of 6-week-old mice (Charles River, Burlington, MA). At 12 days after implantation, treatments began. The dose of GLSF (2 g/kg) was given by oral gavaging once a day, M-F, for a total of nine oral doses. Mice were treated with vehicle control or GLSF 2.0 g/kg (n = 3–4).

For a rodent diet-based drug regimen study, 4-week-old mice were randomly divided into four groups. Groups 1 and 3 (n = 8) were fed with standard diet while groups 2 and 4 were fed with diet containing GLSF (n = 4) for 4 weeks before cell inoculation. When the mice were 8 weeks old, 5 × 10^5^ cells in 100 µl serum free media were injected subcutaneously into the right and left flanks of mice. The dose for GLSF obtained from diet consumption was estimated to be 2.0 g/kg, as for the pilot study. Groups 3 and 4 were treated with a single i.v. dose of abraxane (20 mg/kg) (on Day 13 after tumor inoculation). The tumor volume was calculated according to the formulation: Volume = (width)^2^ × length/2.

### RNA extraction, library preparation, and next-generation sequencing

Tumors from the rodent diet-based drug regimen study were dissected, snap frozen, and stored at −80 °C. The total RNA was extracted from the tumor tissues using TRIzol reagent (Invitrogen, Grand Island, NY) and a RNeasy Mini Kit (Qiagen, Germantown, MD). The quality of RNA samples was determined with an Agilent 2100 Bioanalyzer. A total of eight RNA samples (four samples per treatment, two groups) were sent to Fulgent Genetics (Temple City, CA) for library preparation and sequencing. Briefly, the library was constructed using the NEBNext® Poly(A) mRNA Magnetic Isolation Module + NEBNext® UltraII Directional RNA Library Prep Kit (New England Biolabs, Ipswich, MA). The RNA-seq libraries were sequenced using an Illumina HiSeq 4000 with 150 bp, paired-end, at a minimum depth of total 60 million reads per sample.

### Computational RNA-seq data analysis

Raw data were converted into fastq files by Illumina bcl2fastq2 v2.20. Read quality was assessed using FastQC. The sequence reads were mapped to mouse reference genome GRCm38 using the Edico Genome Dragen aligner with default settings. Duplicate reads, as marked by the Dragen aligner, were removed before coverage analysis. The aligned bam files were processed by HTSeq for gene quantification. Only genes with counts per million (CPM) values above 0.5 in at least two samples were included in the differential expression analysis. Differentially expressed genes were identified by R package EdgeR (version 3.24.3). Briefly, read counts were fitted into negative binomial distribution. The differential analysis was carried out using quasi-likelihood F-test. Genes with *P* values <0.05 and absolute log 2-fold change above 1 were considered to be significantly differentially expressed. Pathway analysis and prediction based on significant DEGs were performed using Ingenuity Pathway Analyses (IPA; Ingenuity® Systems, www.ingenuity.com). Global MDS, hierarchical clustering heat map, and volcano plot were generated using plotMDS, heatmap.2, and plotMD functions in R, respectively.

### Statistical analysis

Data are expressed as mean ± standard deviation unless stated otherwise. All plots were made using GraphPad Prism version 7.0 (GraphPad Software, Inc, La Jolla, CA), and statistical analysis was conducted using NCSS 2007 (NCSS, LLC, Kaysville, UT). The specific tests are detailed in the text and figure legends. For all the statistical analysis, means were indicated to be statistically different when *P* < 0.05.

## Results

### Identification of optimal extraction methods for GLSF

As there is no standardized preparation approach for GL and related products, we tried several methods for preparation of GLSF extracts, including the use of ethanol, methanol, hot water, as well as artificial gastrointestinal juice. To compare the pharmacologic activity of these extracts, we examined their ability to inhibit cancer cell growth *in vitro*. The extracts were examined on a panel of cancerous and non-cancerous cell lines derived from different tissues of origin (PC3, MDA-MB-231, A375, H460, HT-29, HepG2, NCI-N87 and NHDF) using a sulforhodamine B (SRB) colorimetric assay. Comparison of the cytotoxicity data for the four extracts in melanoma A375 or breast cancer MDA-MB-231 indicated that the gastrointestinal juice and hot water extracts exhibited relatively higher potencies of cancer cell growth inhibition (Supplementary Figs. S1 and S2). The viability data and IC_50_ values in these cell lines for gastrointestinal juice and hot water extracts are summarized in Supplementary Table S1. Additionally, our previous study showed higher contents of triterpenes from GLSF in the gastrointestinal juice extract compared to the chloroform extract.^22^ As the gastrointestinal juice extract showed the highest potency on the majority of cancer cell lines, it was selected for chemical fingerprint analysis and examined for *in vitro* anticancer effects.

### Chemical fingerprint analysis of gastrointestinal juice extract of GLSF by HPLC

Based on a literature search for components of GL products, 13 compounds were selected as chemical markers for GLSF. A HPLC-DAD fingerprint method has been developed^22^ and was used to quantify these markers in the extract of GLSF. The contents of 13 compounds are shown in Supplementary Table S2. Among the 13 assessed compounds, only ganoderol A was not detectable; the other 12 compounds were identified with substantial amounts. The inter-batch variation was below 16.5% for all these compounds. Although the chemical markers mainly consisted of triterpenes (polysaccharides not included), the results indicated that at least for selected active components GLSF was efficiently and consistently extracted.

### Effects of GLSF extract on chemosensitivity of colorectal cancer

**Figure 1.**
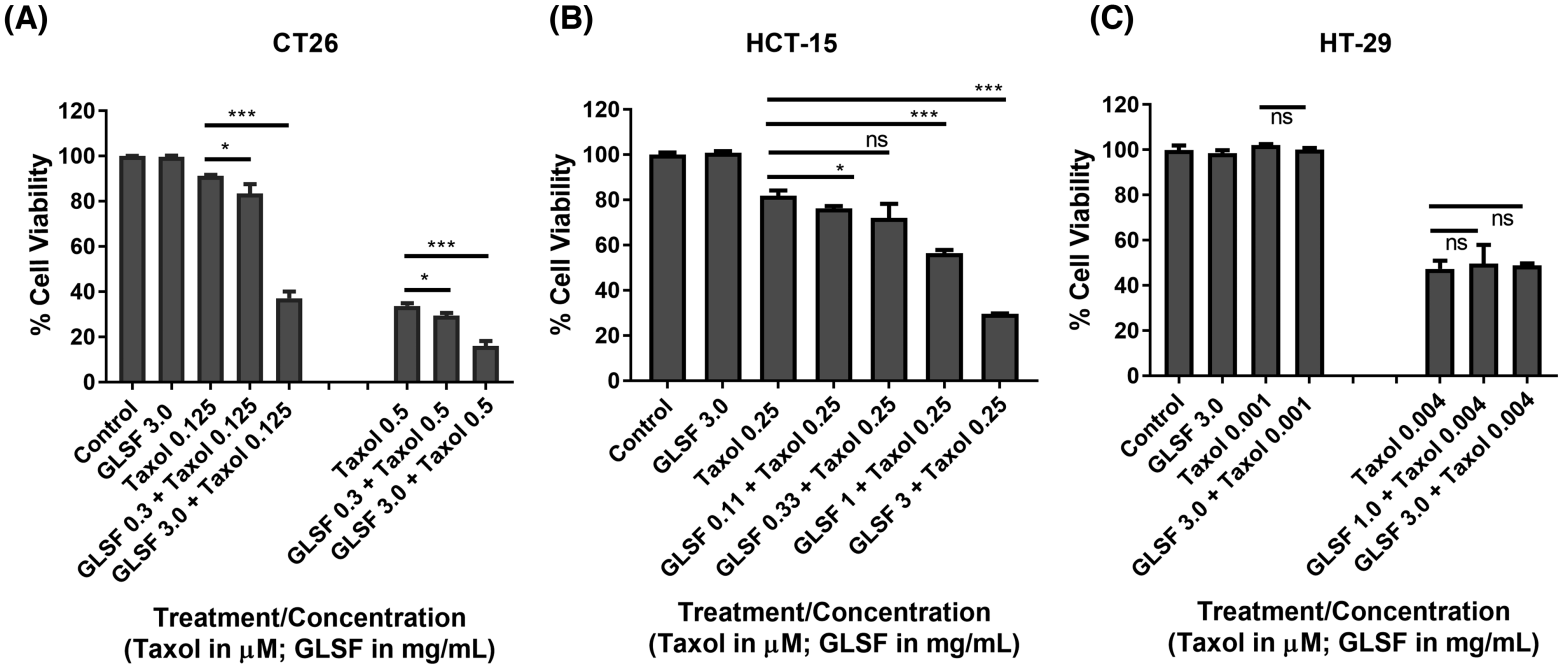
Effects of GLSF gastrointestinal (GI) juice extract on the growth inhibitory effects of paclitaxel in colorectal cancer cells. (A) SRB cytotoxicity assay was used to determine the effects of paclitaxel, GLSF, alone or in combination in the mouse colon cancer CT26 cells. (B) The cytotoxicity of paclitaxel, GLSF, alone or in combination in human colon cancer HCT-15 cells. (C) The effects of paclitaxel, GLSF alone or in combination in human colon cancer HT-29 cells. *: *P* < 0.05, ***: *P* < 0.001, comparing groups treated with paclitaxel alone with other groups, as determined by *t* test.

Paclitaxel (taxol) exhibited a modest inhibitory effect on CT26 cells (IC_50_ was 0.45 ± 0.02 μM), while GLSF did not cause cytotoxicity in CT26 at concentration up to 3 mg/ml. However, the cells that were co-treated with taxol (0.125 μM) and GLSF (0.3 mg/ml) showed significantly increased cytotoxic effects compared to the taxol treatment alone (*P* < 0.05) (Fig. 1a). We observed a dose-dependent chemosensitizing effect of GLSF, where a higher concentration (3.0 mg/ml) showed an increased chemosensitizing effect compared to GLSF at 0.3 mg/ml (*P* < 0.001). Similar effects were observed for combined treatment of GLSF (0.3 or 3.0 mg/ml) with a higher concentration of taxol (0.5 μM). To confirm the nature of the interaction between GLSF and taxol, combination analyses were performed with the Combination Index (CI) method,^23,24^ using drug combinations at various ratios. A synergistic interaction was produced (CI = 0.04) when taxol 0.125 μM was combined with GLSF at 3.0 mg/ml (CI values < 1 indicate synergistic activity). To confirm these results in human colorectal cancer cell lines, SRB assays were performed on human colorectal cancer HCT-15 and HT-29 cells, treated with taxol and GLSF alone or in combination. The IC_50_ in HCT-15 treated with taxol was 0.40 ± 0.002 μM and similar to the CT26 cells, GLSF did not cause any cytotoxicity in HCT-15 cells up to 3 mg/ml concentration. The cells that were co-treated with taxol (0.25 μM) and GLSF (0.11–3 mg/ml) showed a dose-dependent increase in cytotoxic effects compared to the taxol treatment alone (*P* < 0.05 for GLSF 0.11 mg/ml; *P* < 0.001 for GLSF 1.0 and 3.0 mg/ml) (Fig. 1b). Although HT-29 cells were much more sensitive to taxol (IC_50_ = 0.0035 ± 0.001 μM) than CT26 and HCT-15 and GLSF at 3 mg/ml alone was also nontoxic, co-treatment with GLSF and taxol did not demonstrate increased or decreased effect on cell viability (Fig. 1c). Therefore, the chemosensitizing effect for GLSF is variable in different cancer cell lines.

### Effects of GLSF extract on taxol-induced apoptosis in CT26 cells

**Figure 2.**
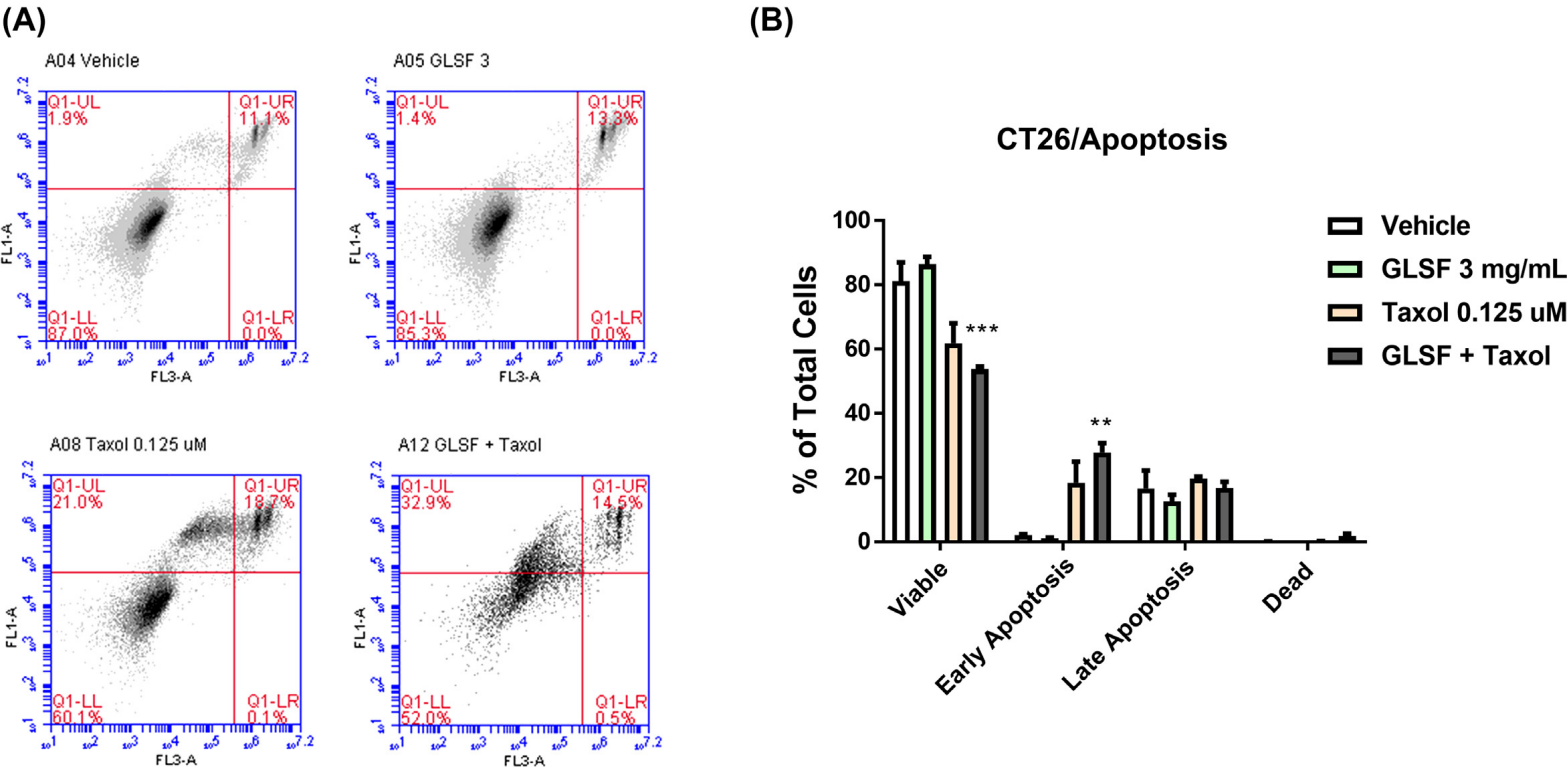
Effects of GLSF GI juice extract on apoptosis induced by paclitaxel in mouse colorectal cancer CT26 cells. (A) Cell apoptosis was assessed by Annexin V/PI double staining which was detected by flow cytometry FACS analysis; Annexin-V-FITC staining in y axis (FL1) and PI in x axis (FL3). (B) The Annexin V/PI assay was performed three times and the average percentage of viable, early apoptosis, late apoptosis, and necrosis populations of cells were plotted. **: *P* < 0.01, ***: *P* < 0.001, compared with vehicle control group, as determined by *t* test.

The degree of apoptosis induced by GLSF (3 mg/ml), taxol (0.125 μM), or their combination was evaluated in CT26 cells. The cells were incubated for 72 hours with combinations of drugs and the level of apoptosis was quantified by an Annexin-V binding and PI staining assay. As shown in Fig. 2, GLSF treatment slightly increased the viable cells compared to vehicle control (*P* > 0.05). Taxol decreased the viable cell population to 60.1 ± 6.2% (*P* > 0.05). When the cells were exposed to a combination of GLSF and taxol, there was a significant reduction of viable cells (*P* < 0.001) (53.7 ± 1.6% of viable cells in control) and increase in the number of early apoptotic cells (*P* < 0.01). This result is consistent with those data obtained by SRB in CT26 cells (Fig. 1a), indicating that the co-treatment with non-toxic concentration of GLSF increased the anticancer potency of taxol via enhancing apoptosis in CT26 cells.

### Effects of GLSF extract on P-glycoprotein (MDR1) activity in K562/DOX cells

**Figure 3.**
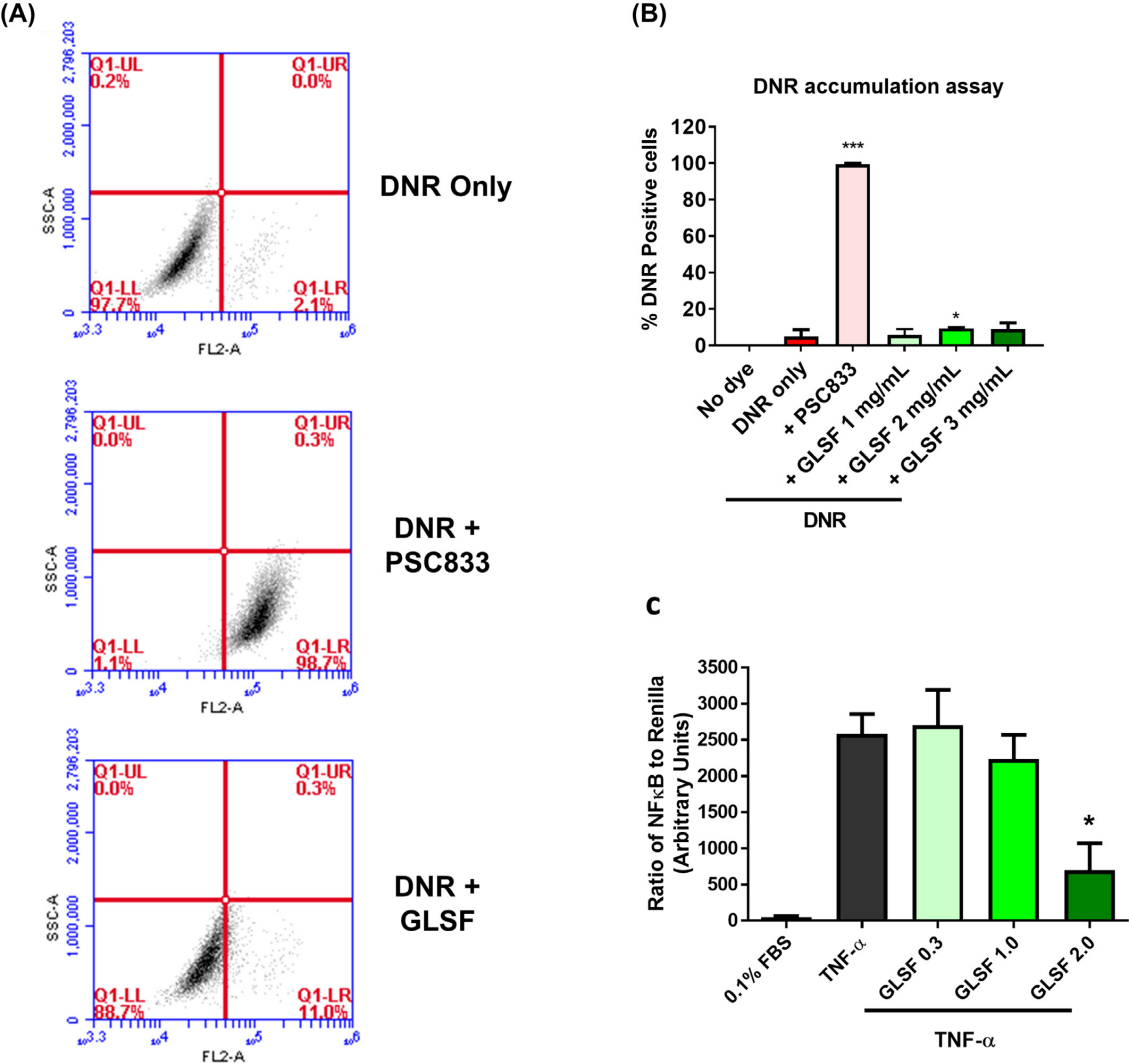
Effects of GLSF GI juice extract on P-glycoprotein activity in K562/DOX cells and NF-κB activity in HEK-293 cells. (A) Flow cytometry analysis of DNR accumulation in cells incubated with DNR, treated with or without PSC833 (10 μM) or GLSF. Representative flow cytometry data are shown. (B) Graphs showing percentage of cells that were positive for DNR in cell samples co-treated with GLSF and DNR in comparison with those co-treated with 10 μM PSC833 and DNR. The data are presented as the mean ± SD from 3–6 independent experiments. *: *P* < 0.05 compared to the DNR only group. (C) Effects of GLSF on TNF-α-induced NF-κB promoter activity. HEK-293 cells transfected with NF-κB luciferase reporter and *Renilla* control reporter were treated with vehicle, TNF-α (10 ng/mL), GLSF (0.3, 1.0, 2.0 mg/mL) combined with TNF-α for 5 hours. Data are expressed as mean +/- SEM; n = 3–6. *: *P* < 0.05 as per one-way ANOVA.

Previous studies have shown that both CT26^25^ and HCT-15^26^ express the efflux transporter P-glycoprotein (P-gp or MDR1, *ABCB1* gene), which is part of the mechanism for their intrinsic resistance to taxanes and other P-gp substrate drugs such as daunorubicin (DNR). Therefore, we examined whether GLSF was able to inhibit P-gp-mediated drug efflux from the multi-drug resistant cell line K562/DOX, which overexpresses P-gp, using daunorubicin (DNR) as a fluorescent substrate.^20^ Few cells were positive for DNR accumulation without P-gp inhibitor (Fig. 3a). Co-treatment with a known P-gp inhibitor PSC833 (10 μM) as a positive control greatly increased DNR positive cells to nearly 100%. However, GLSF (1, 2 and 3 mg/ml) only slightly increased DNR accumulation (Fig. 3b). Although the treatment effect of GLSF (2 mg/ml) was significant (*P* < 0.05), compared to the positive control PSC833, the degree of inhibition was negligible. This result suggests that the chemosensitizing activity of GLSF is not attributed to P-gp inhibition.

### Effects of GLSF extract on NF-κB promoter activity in HEK-293 cells

We next evaluated the effect of GLSF on TNF-α promoted NF-κB activation. The HEK-293 cells were transfected with NF-κB-luc firefly luciferase reporter construct and a plasmid encoding the *Renilla* luciferase for the dual luciferase assay. The transfected cells were treated with vehicle (control) or GLSF extracts at 0, 0.3, 1.0, or 2.0 mg/ml, followed by a 5-hour co-incubation with TNF-α (10 ng/ml). As expected, TNF-α strongly stimulated NF-κB activity (Fig. 3c). The promoter activity stimulated by TNF-α was inhibited significantly by the GI extract at 2.0 mg/ml in a dose-dependent manner (Fig. 3c). As NF-κB plays a role in promoting carcinogenesis of various types of cancer^27^ and conferring chemoresistance to paclitaxel,^28^ inhibition of NF-κB by GLSF might partly explain the *in vitro* chemosensitizing activity and the *in vivo* activity described below.

### Anticancer efficacy of GLSF oral administration to mice bearing murine colorectal cancer tumor CT26

To evaluate the anticancer activity of GLSF *in vivo*, a syngeneic model of colorectal cancer-bearing BALB/c mice was used. We firstly conducted a pilot study using a small number of mice with two tumors (CT26) implanted in each mouse (n = 2 each gender specific group).

The treatment by oral gavage (2.0 g/kg, daily, Monday to Friday, nine doses in total) was started on the 11th day after tumor implantation. Although statistically insignificant, a trend of treatment effect was observed in tumor volume in both males and females (Supplementary Fig. S3): the average tumor volumes in GLSF-treated mice were smaller than in untreated control. In addition, the mice did not lose body weight compared to initial weight, indicating the treatment was not toxic.

To explore the potential anticancer mechanism, the effect of GLSF treatment on mouse spleen lymphocyte proliferation induced by concanavalin A (Con A) or lipopolysaccharides (LPS) *in vitro* was determined using the spleen samples obtained from the CT26 tumor-bearing mice. Compared with non-tumor-bearing mice, proliferation of spleen T and B lymphocytes induced by Con A and LPS were strongly declined in tumor-bearing mice (Supplementary Fig. S3). However, treatment with GLSF of these mice increased spleen T and B lymphocyte proliferation in CT26-bearing mice, although this was not statistically significant. These data indicate a potential immunomodulatory activity of GLSF, consistent with a previous report.^4^

**Figure 4.**
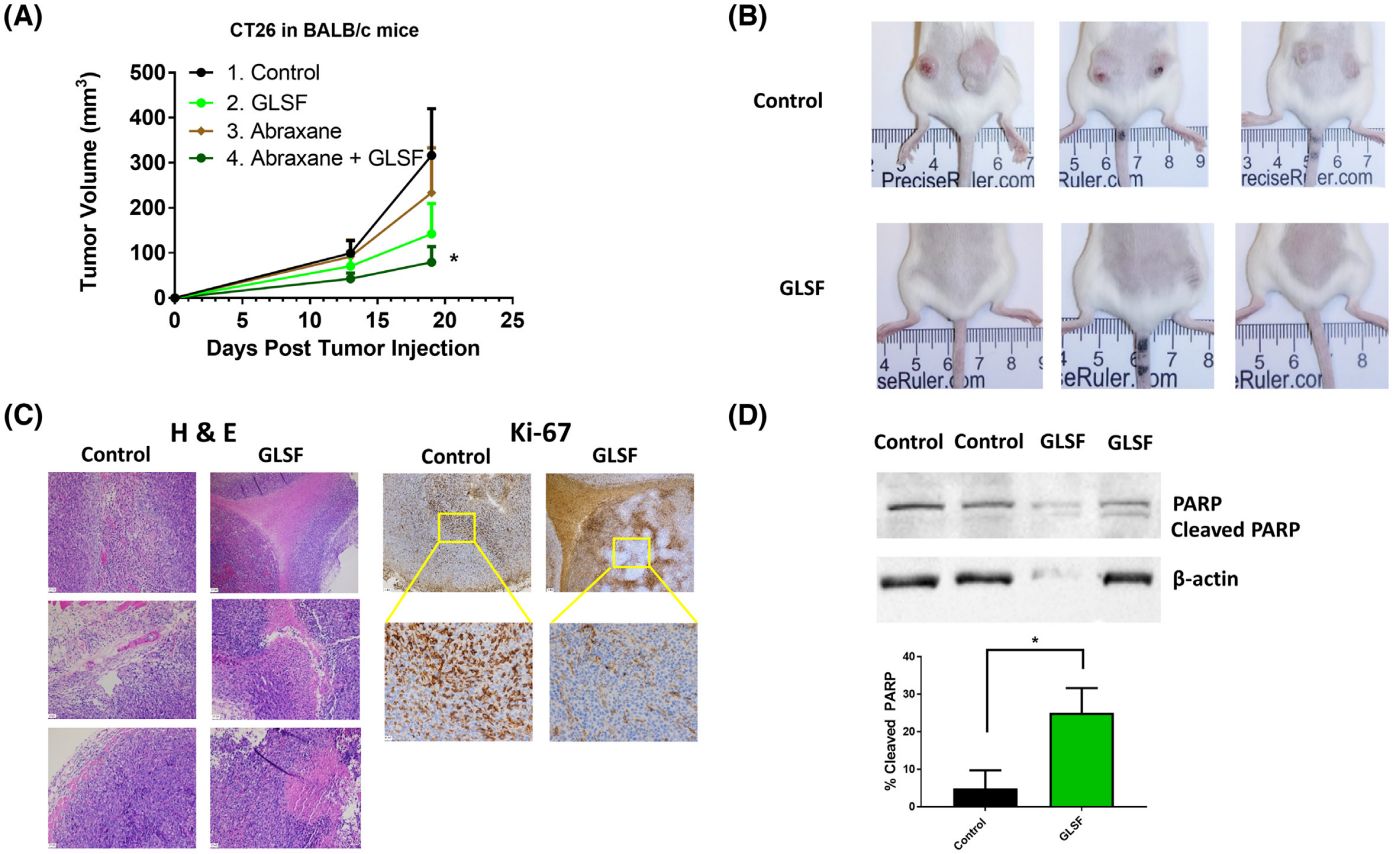
Effects of GLSF on tumor growth and apoptosis in a colorectal cancer syngeneic model. (A) The CT26 cells were subcutaneously implanted into the female BALB/c mice after the mice were fed with the control diet (groups 1 and 3) or diet containing GLSF (groups 2 and 4) for 4 weeks. Groups 3 and 4 mice were treated with a single dose of abraxane® on Day 13. Tumor volumes were measured on Days 13 and 19 (Day 0 was the day of tumor injection). *: *P* < 0.05 by Tukey-Kramer Multiple-Comparison test (n = 8 for G1 and G2; n = 4 for G3 and G4). (B) Representative photos of mice in control and GLSF groups. (C) Tumor tissues were stained with H&E or immunohistochemical staining of Ki-67. (D) Western blot analysis of PARP, cleaved PARP, and β-actin. Representative gel images are shown. Bar graphs represent the ratios of cleaved PARP/total PARP. *: *P* < 0.05 as compared with the control group by *t* test. PARP, poly (ADP-ribose) polymerase. Control n = 5 tumors, GLSF n = 4 tumors.

To confirm the anticancer activity of GLSF, BALB/c mice were pre-treated with control diet (groups 1 and 3) or modified diet containing GLSF (groups 2 and 4) 1 month before tumor implantation. As GLSF showed antitumor effects in both genders in the pilot study and the CT26 tumor was derived from female mice,^15^ only females were included in this experiment. Abraxane treatment was given for groups 3 and 4 to observe the effects of combination treatments. Two tumors were implanted in each mouse. Consistent with the pilot study, GLSF treatment alone inhibited tumor growth (Fig. 4a), although repeated measures ANOVA analysis did not show a statistically significant difference. At the end of the experiment, 100% of the mice had two tumors in the control group, while only 63% of mice in the GLSF group had two tumors, and two of the eight mice (25%) in the GLSF group did not show any tumors (Fig. 4b). The Tukey-Kramer *post hoc* analysis showed that on Day 19 after tumor inoculation, co-treatment with abraxane and GLSF significantly suppressed tumor growth compared with the control group (Fig. 4a). Furthermore, tumors in the control group (group 1) showed a significant increase when comparing tumor size on Day 13 and 19, while groups 2, 3 and 4 did not show time-dependent tumor size increase, confirming the treatment effect.

Tumor tissues dissected from the control and GLSF groups were subjected to hematoxylin and eosin (H&E) staining. The image of H&E sections indicated that tumor tissues from both groups consisted of a morphologically similar differentiated adenocarcinoma. However, tumors in the GLSF group showed a conspicuous increase of necrosis in all the four tumor samples submitted for H&E staining (representative images are shown in Fig. 4c). Ki-67 staining showed that the tumors in the control group consisted entirely of one cell type with high proliferative index, while the tumors in the GLSF group demonstrated nonspecific staining of necrotic/apoptotic cells but showed fainter staining of fewer Ki-67 positive viable cells (Fig. 4c).

Western blot analysis was used to examine the expression of the apoptosis marker PARP and cleaved PARP in tumor tissue lysates. The expression of cleaved PARP was markedly increased in the GLSF treatment group compared with that in the control group (Fig. 4d), and this difference was statistically significant (*P* < 0.05), confirming that GLSF treatment induced apoptosis in tumor tissues *in vivo*.

### RNA-seq profiling of GLSF-treated tumors

**Figure 5.**
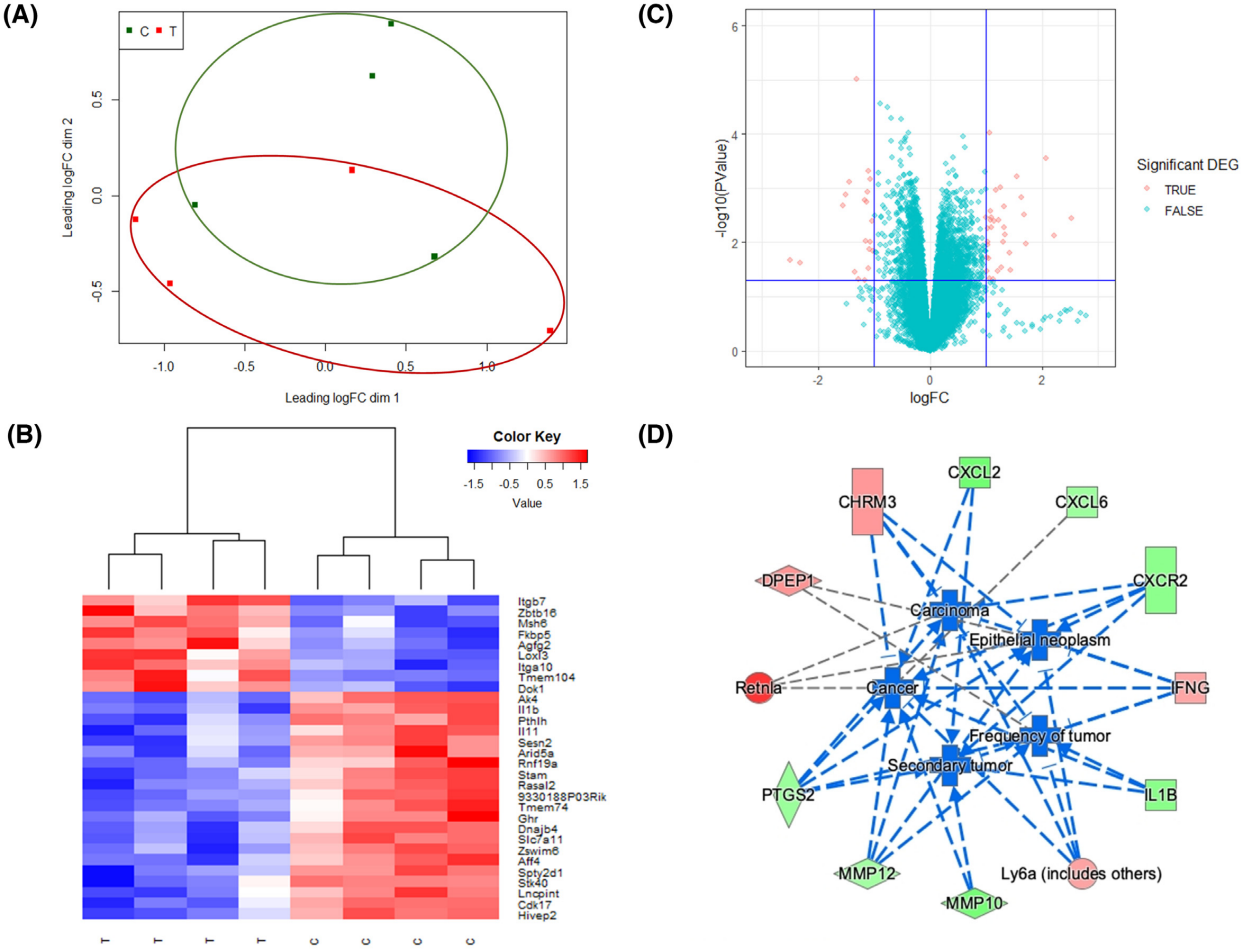
RNA-seq analysis of CT26 syngeneic tumors in mice treated with control diet or GLSF-modified diet. (a) Multi-dimensional scaling plot of detected genes in GLSF treatment (T, red) and control group (C, green). The distances correspond to leading log2 fold-changes between each pair of samples. (b) Volcano plots of differentially expressed genes between treatment and control groups. The red dots indicate up- and down-regulated DEGs with *P* < 0.05 and absolute log2 FC > 1. (c) Heat map of top 30 significantly differentially expressed genes between treatment (T) and control groups (C). (d) Genes that are up- and down-regulated in treatment group (compared to control) are displayed within red or green nodes, respectively. The predicted inhibited biology effects are presented in blue nodes. Blue (predicted to be inhibited) or gray (undetermined direction) dash lines represent relationships with causal consistency.

To explore the mechanism underlying the treatment effects by GLSF, RNA-seq analysis was performed to compare the gene expression profiles between tumors isolated from mice subjected to the control diet (n = 4) or GLSF modified diet (n = 4). On average, 40 million pair-end reads pairs per sample were generated, with all of their uniquely mapping rates above 91%. Overall, RNA sequencing detected 25  051 genes across eight samples, of which 13  790 genes had a CPM value above 0.5 in at least two samples. A multi-dimensional scaling (MDS) plot showed a trend of clustering separation between treated group and control group (Fig. 5a). Out of the 13 790 genes, 53 were identified as significant differentially expressed genes (DEGs) between the two groups with a absolute log2 fold-change >1 and a *P* value <0.05 (Supplementary Table S3). The top 12 up-regulated genes and top 10 down-regulated genes (selected based on the lowest *P* value) are presented in Tables 1 and 2. A log2 fold-change lower than −1 indicated that the expression of the gene was decreased by GLSF treatment, whereas a log2 fold-change more than 1 suggested an increased gene expression in tumor tissues derived from GLSF treatment. A heat map of the top 30 significant DEGs was plotted with unsupervised hierarchical clustering (Fig. 5b). A volcano plot was generated with the significant DEGs highlighted in red color based on their *P* values and fold changes (Fig. 5c). It is notable that many of these GLSF down-regulated genes are involved in NF-κB-regulated inflammation, such as IL-1β (*Il1b*) and IL-11 (*Il11*) (Table 2, Fig. 5b).

**Table 1.** Top 12 up-regulated genes in tumors from mice treated with GLSF compared to control group.

Gene ID	Gene name	Log FC	*P* value
*Itga10*	Integrin, alpha 10	1.07	9.34E-05
*Zbtb16*	Zinc finger and BTB domain containing 16	2.07	2.79E-04
Gm807	Predicted gene 807	1.26	9.47E-04
*Lvrn*	Laeverin	1.17	1.07E-03
Gm6093	Predicted gene 6093	1.63	1.49E-03
Hrct1	Histidine rich carboxyl terminus 1	1.33	2.15E-03
*Aldh1a1*	Aldehyde dehydrogenase family 1, subfamily A1	1.08	2.59E-03
*Inpp5j*	Inositol polyphosphate 5-phosphatase J	1.67	3.06E-03
*Rcan2*	Regulator of calcineurin 2	1.00	3.39E-03
*Ifng*	Interferon gamma	1.09	3.52E-03
*Retnla*	Resistin like alpha	2.52	3.63E-03
*Fmo2*	Flavin containing monooxygenase 2	1.35	7.23E-03

* Log FC (log2 Fold change) larger than 1 indicates higher expression in GLSF-treated tumors.

**Table 2.** Top 10 down-regulated genes in tumors from mice treated with GLSF compared to control group.

Gene ID	Gene name	Log FC	*P* value
*Il1b*	Interleukin 1 beta	−1.31	9.57E-06
*Il11*	Interleukin 11	−1.09	4.89E-04
4930565N06Rik	RIKEN cDNA 4930565N06 gene	−1.06	6.84E-04
*Nppb*	Natriuretic peptide type B	−1.44	7.72E-04
*Ptgs2*	Prostaglandin-endoperoxide synthase 2 (Cox-2)	−1.11	1.18E-03
*Mmp10*	Matrix metallopeptidase 10	−1.51	1.31E-03
*Mmp13*	Matrix metallopeptidase 13	−1.16	1.68E-03
*Stamos*	Signal transducing adaptor molecule (SH3 domain and ITAM motif) 1, opposite strand	−1.13	1.82E-03
*Cxcl1*	Chemokine (C-X-C motif) ligand 1	−1.56	2.07E-03
*Mmp12*	Matrix metallopeptidase 12	−1.03	4.01E-03

* Log FC (log2 Fold change) smaller than −1 indicates lower expression in GLSF-treated tumors.

To explore the possible biological functions linked with these DEGs, ingenuity pathway analysis (IPA) was performed to identify canonical pathways, upstream regulators, diseases and functions that are associated with GLSF treatment. Based on the ratio of the number of DEGs in our dataset to the total number of reference genes in the specific pathways in the IPA knowledge bases, a Fisher's exact test was used to determine the canonical pathways associated with the treatment effect. Using a cutoff *P*value <0.05, a total of 58 canonical pathways were identified as being significantly enriched based on the DEGs (Supplementary Table S4). The most affected pathways were “Granulocyte adhesion and diapedesis” and “Agranulocyte adhesion and diapedesis”, which are known immune/inflammatory pathways.

Upstream analysis through IPA was used to predict the upstream regulators potentially causing changes in gene expression and regulation direction based on the DEGs. Predicted significantly activated and inhibited regulators are listed in Supplementary Table S5 (*P* value < 0.05, |Z-score|>2). The two significantly activated upstream regulators were *alpha*-catenin and TP53, both of which are known tumor suppressors for colon cancer. On the other hand, the most inhibited upstream regulators were factors that play critical roles in inflammatory response and tumorigenesis, including TNFSF12, KRT17, IL17RA, TNF, and IL17A.

**Figure 6.**
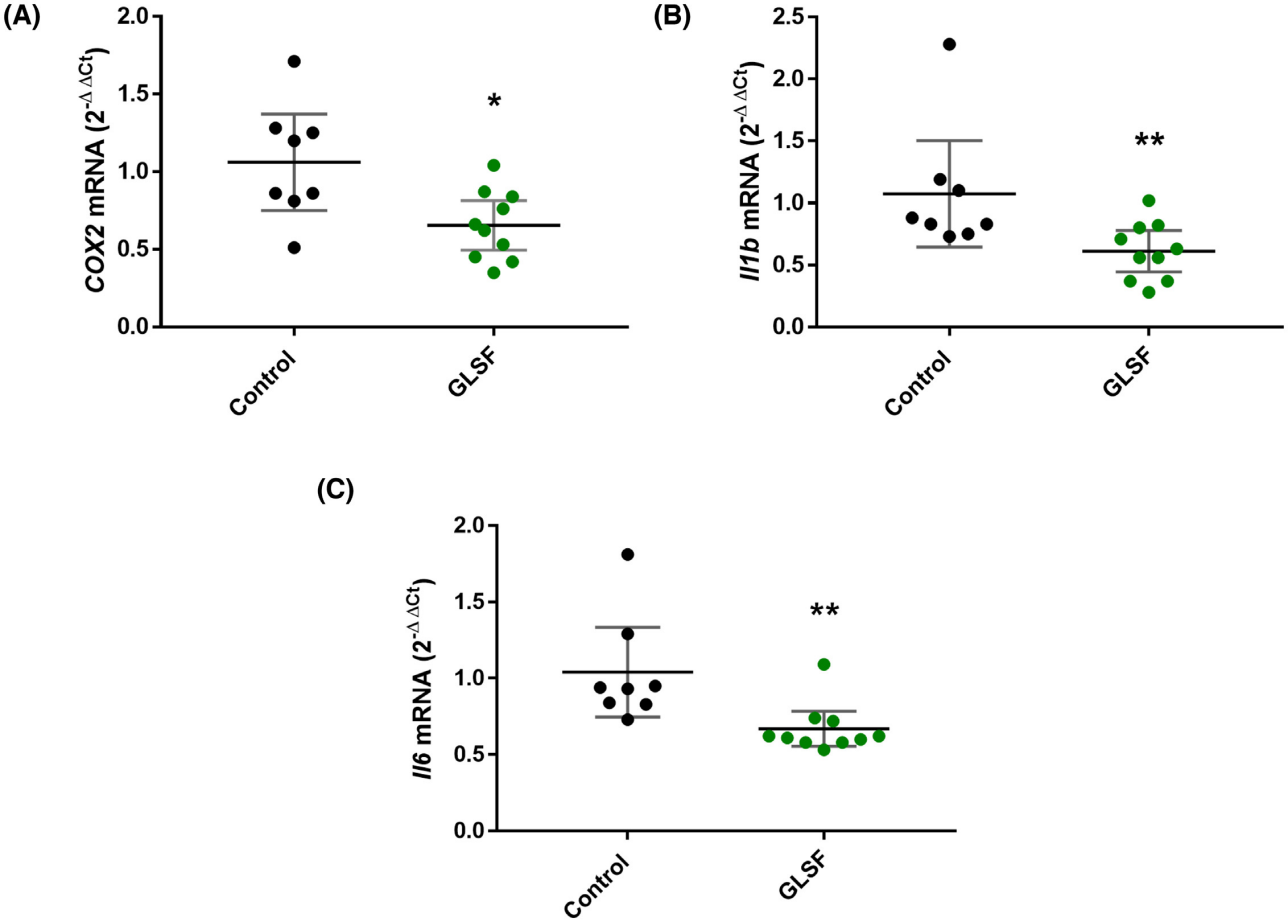
qRT-PCR results from tumor samples of control or GLSF groups for (A) COX-2, (B) IL-1β, and (C) IL-6. β-actin was used as normalization control and data are expressed as mean with 95% CI. n = 8 tumor samples from control group. n = 10 tumor samples for GLSF. *: *P* < 0.05; **: *P* < 0.01 via a Mann-Whitney U approximation without correction for normality.

"Diseases and functions" in IPA was used to predict affected biology and its regulation direction associated with GLSF treatment. Using a cutoff of *P* value < 0.05, |Z-score|>2, there was no significantly activated biology effect identified, while the top five significantly inhibited biological effects are presented in Fig. 5d, which included “Cancer”, “Epithelia neoplasm”, “Carcinoma”, “Secondary tumor”, “Frequency of tumor”. The network of the top five inhibited biology effects in the GLSF treatment group, compared to the control, and the contributing DEGs were displayed in Fig. 5d. Quantitative RT-PCR analysis confirmed that GLSF inhibited the expression of selected inflammatory genes, cyclooxygenase-2 (*Ptgs2*or *Cox-2*) (*P* < 0.05), interleukin-1β (*Il1b*) (*P* < 0.01), and interleukin-6 (*Il6*) (*P* < 0.01) (Fig. 6).

## Discussion

The present study examined the *in vitro* and *in vivo* anticancer activity of GLSF, which contains a mixture of broken spores and fruiting bodies of mushroom. As different methods of extraction affect the release, bioavailability and pharmacological activity of active components, extracts of GLSF were prepared in ethanol, methanol, hot water, or artificial gastrointestinal juice and were examined in a panel of cell lines. We found that extracts prepared using gastrointestinal juice had the highest potency of cell growth inhibition and therefore were used in our *in vitro* studies. This extraction method is physiologically relevant to clinical application. Although the method of extraction has been used for ginseng^29^ and notoginseng (“San-Qi”),^30^ to our knowledge, this is the first study using artificial gastrointestinal juice to prepare GL extracts. *In vitro*, GLSF alone showed modest cytotoxicity on colon cancer cells, which is consistent with a previous study indicating that the direct anticancer effect of GL is limited.^3^ However, when GLSF was combined with taxol, it induced stronger tumor inhibition and apoptosis, suggesting that GLSF may be used as a chemosensitizer (Fig. 1a and b). As GLSF is not a P-gp inhibitor (Fig. 3a and b), its chemosensitizing mechanism remains to be determined. One possible mechanism is through its inhibitory activity against NF-κB signaling, suggested by the dual luciferase assay (Fig. 3c). Consistently, previous studies have shown that triterpene ganoderic acid C1 isolated from GL inhibited inflamed Crohn's disease colonic mucosa as a result of blockage of NF-κB.^31^

*In vivo*, GLSF alone or in combination with abraxane (nanoparticle albumin bound paclitaxel or nab-paclitaxel) induced tumor growth inhibition and apoptosis (Fig. 4). The syngeneic tumor model used in the study was murine colorectal carcinoma cell line Colorectal Tumor #26 (CT26).^15^ CT26 cells were derived by exposing BALB/c mice to the chemical carcinogen N-nitroso-N-methylurethane, resulting in a rapid-growing grade IV carcinoma that is easily implanted and readily metastasizes.^32^ As CT26 tumor in the immunocompetent BALB/c mice provides a syngeneic model, it is frequently used for developing and testing immunotherapeutic agents.^15,33^ Compared with *in vitro* data, a large *in vivo* effect suggests that an intact immune system or tumor microenvironment may be essential for the pharmacological action of GLSF. One limitation for these *in vivo* studies was that a single dose was used (2.0 g/kg), which was derived from published data of GL.^34–36^ Dose-response studies should be considered in the future. Although taxol or abraxane are effective to many types of cancer such as breast or ovarian cancer, their application in colorectal cancer has been limited because of intrinsic resistance.^37^ Thus, GLSF may be used as a chemosensitizer for taxanes. The ratio of GLSF versus taxol or abraxane should be optimized in future studies to identify the optimal combinational regimen. Further studies comparing therapeutic effects of GLSF in different tumor models and with other anticancer agents should be considered.

To gain mechanistic insight, RNA-seq was performed to evaluate genome-wide transcriptome changes associated with GLSF treatment. Based on the differentially expressed genes (DEGs), expression levels of IL-1β and IL-11, genes encoding for cytokines in the tumor microenvironment that promote colorectal cancer progression, were decreased by GLSF. These cytokines, also including Il-6 which was not picked up by RNA-seq analysis because of low CPM values, are produced by myeloid and T-helper interleukin (IL)-17-producing (Th17) cells that are accumulated in the tumor microenvironment.^38^ Because these cytokines directly or indirectly activate neoplastic epithelium, therapies that target their activation, for example anti-IL-11 therapy, have been proposed to treat colorectal cancer.^38^ In addition, the GLSF down-regulated *Ptgs2*, encoding for Cox-2, known to be an inflammatory mediator that promotes colorectal cancer.^39^ RT-PCR results confirmed that in GLSF-treated mice the tumor cell expression of Cox-2, IL-1β, and IL-6 was significantly down-regulated at mRNA level (Fig. 6). These results suggest that inflammation is a potential target for treatment of some types of colorectal cancer.^40^ Consistent with the luciferase assay data (Fig. 3c), these GLSF-down-regulated genes are under the control of NF-κB transcription factor. Furthermore, CT26 cells harbor the constitutively activating mutant *KRAS* ,^15^ which has been shown to trigger the production of several inflammatory mediators including IL-6,^41^ IL-1β^42^ and associated with COX-2.^43^ Interestingly, NF-κB is activated in *KRAS*-mutated cancer and has been suggested as a target to treat *KRAS*-induced cancer.^44,45^ In addition, a previous study showed that CT26 cells not only secret IL-6, but also the growth of CT26 tumor depends on IL-6.^46,47^ Thus, these inflammatory factors, many of which are under the regulation of NF-κB, are very likely targets of a GL-mediated anticancer effect. These results also suggest that inflammation-associated cancers and KRAS-driven tumors might be more likely to respond to GLSF. As inhibiting KRAS directly is very challenging, approaches to disrupt downstream signaling pathways may be a better approach to treat these types of highly aggressive cancer.^48^

Another down-regulated gene in the GLSF-treated group was the inflammatory chemokine *Cxcl1*, which is involved in enhanced metastatic potential of colon cancer by increasing cell migration, matrix metalloproteinases (MMP) expression, and epithelial-to-mesenchymal transition and therefore has a negative prognostic impact to the clinical outcome.^49^ Probably associated with *Cxcl1* down-regulation, three MMP genes were among the top 20 down-regulated genes: *MMP10, MMP13*, and *MMP12*. These genes and their products have been identified as negative prognostic markers in colon cancer patients.^50,51^ GLSF down-regulated *Nppb*, encoding for natriuretic peptide B, which has been shown to be a key oncogene candidate for colon tumors and suggested as one of the early biomarkers for prevention in the clinical setting.^52^

The GLSF-up-regulated genes have various functions. Although most of these genes have not yet been associated with colon cancer, they may contribute to GLSF-induced anti-cancer activity. Some of them may be tumor suppressor genes, for example, *Fmo2* (flavin containing monooxygenase 2) plays a role as a tumor suppressor in lung cancer.^53^ The lower level of *Inpp5j*, which encodes inositol polyphosphate-5-phosphatase J, has been associated with more aggressive tumors and poorer survival of cutaneous squamous cell carcinoma,^54^ and was found to be deficient in oropharyngeal squamous cell carcinoma.^55^  *Itga10*, a top DEG of the GLSF-up-regulated genes, encodes integrin subunit α 10, which binds to collagen and plays a role in cell adhesion and cell-surface mediated signaling. Although the activity of increased expression of *Itga10* by GLSF is unknown, the interaction of integrin and collagen may mediate an anti-tumor immune response.^56^

The canonical pathway analysis highlighted “Granulocyte adhesion and diapedesis” and “Agranulocyte adhesion and diapedesis” as the most significantly regulated pathways influenced by GLSF treatment. Both pathways contain the same set of genes, mainly MMPs, and have been associated with immunity and inflammation,^57^ as well as tumor invasion and metastasis.^58^ These results support the notion that these two pathways were involved in inflammation associated with colorectal cancer, as previously reported,^59^ and may contain the molecular targets that underlie the anticancer effect of GLSF. Based on the DEGs between the treatment and control groups, IPA predicted the upstream regulators and their activation states which might result in these gene expression changes. Several inflammatory cytokines were at the top of the inhibition list such as TNFSF12, TNF, and IL17A. On the other hand, tumor suppressors such as alpha-catenin and TP53 were activated (activation z-score > 2) according to the prediction. This prediction further implied that GLSF may elicit anti-tumor activity through its anti-inflammatory effects. More specifically, one possible mechanism is that GLSF treatment resulted in an inhibition of the NF-kB pathway, a shared target of the aforementioned predicted upstream regulators, which eventually may lead to tumor suppression. Further studies are required to validate this hypothesis. Based on the IPA prediction, there was no significantly activated biology effect while the top five inhibited biology effects were all related to “Cancer” and displayed the network of tumor suppression induced by GL treatment (Fig. 5d). These results implied a possible mechanism of GL's anti-tumor effect as alleviating the immune suppression in colorectal tumor tissue by promoting the recruitment of anti-tumor immune cells, or by inhibiting immune suppressive cells such as myeloid derived suppressor cells. This hypothesis align well with the observation that GLSF alone showed little cytotoxicity *in vitro* because of a lack of immune system. It is notable that the *in vivo* studies used GLSF powder while *in vitro* assays used extract. Identification of the active components that are responsible for these observed effects requires further investigation.

In conclusion, this study evaluated the anticancer effects and possible underlying anticancer mechanism of GLSF using both human and murine colorectal cancer cell lines, and examined GLSF alone or in combination with a commonly used chemotherapeutic agent paclitaxel. *In vitro* data revealed that although GLSF alone had little anticancer effect, it had a chemosensitization effect in certain colon cancer cells and that NF-κB played a major role in mediating the *in vitro* anticancer effect of GLSF. Immunocompetent mice carrying the syngeneic tumor CT26 were used for investigating the *in vivo* anticancer activity of GL. RNA-seq and bioinformatics analysis, for the first time, indicated that GLSF targeted inflammation and carcinogenesis and confirmed the role of NF-κB as the potential target. Advanced colorectal cancer shows inherent resistance to paclitaxel or related chemotherapeutic agents. With the use of GLSF, a non-toxic natural product, the antitumor effects of chemotherapy may be enhanced. GSLF alone also had anticancer activity *in vivo* through an inhibitory effect against inflammation, NF-kB, and/or KRAS activation.

## Data and material availability

All materials, data, and associated protocols are available to readers without undue qualifications in material transfer agreements. The GLSF is available from the Beijing Tong Ren Tang Chinese Medicine Co., Ltd.

## Supplementary Material

pbab023_Supplemental_File
