# Supplementary material for: Inhibitory activity of medicinal mushroom Ganoderma lucidum on colorectal cancer by attenuating inflammation
Source: Precis Clin Med. 2021 Aug 28;4(4):231–45. doi: 10.1093/pcmedi/pbab023 (PMC8982591; doi:10.1093/pcmedi/pbab023)
Supplement: pbab023_Supplemental_File [file pbab023_supplemental_file.zip › Supplementary Figures_08102021.pdf]

Supplementary Figure

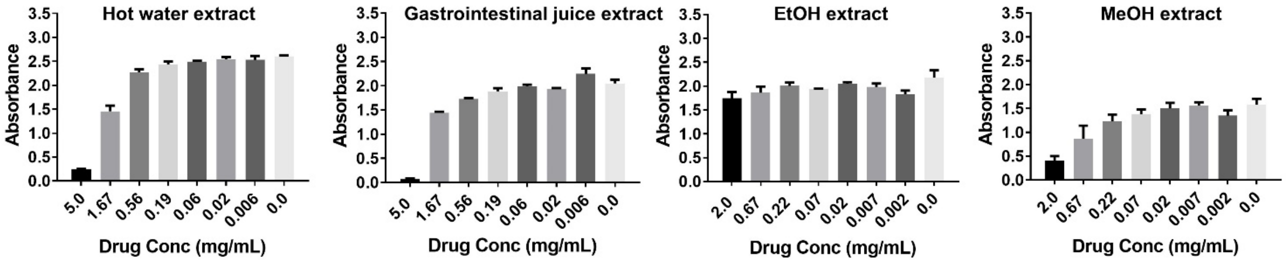

Supplementary Fig S1. Comparison of the cytotoxic effects of various extraction methods for GLSF on melanoma skin cancer cell line A375. SRB cytotoxicity assay was used to determine the effects of GLSF extracts in the human melanoma cell line A375. The cells were treated with extracts in 1:3 serial dilutions with maximum concentrations of 5 mg/mL for hot water and GI extracts or 2 mg/mL for ethanol and methanol extracts. The data shown are the absolute absorbance reading of the plates.

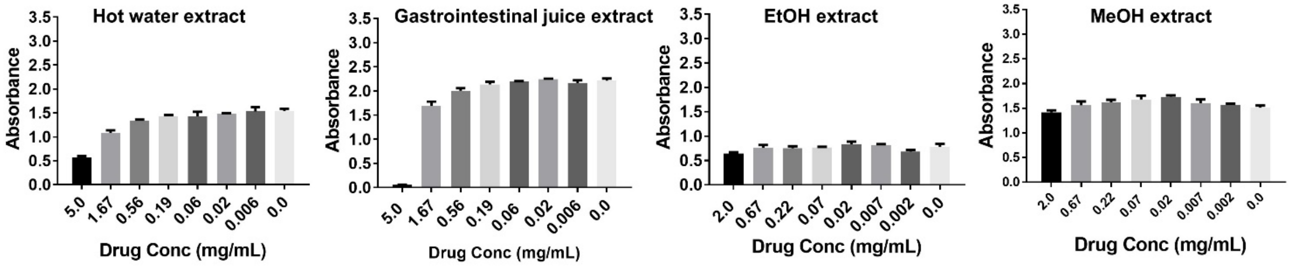

Supplementary Fig S2. Comparison of the cytotoxic effects of various extraction methods for GLSF on breast cancer cell line MDA-MB-231. SRB cytotoxicity assay was used to determine the effects of GLSF extracts in the human breast cancer cell line A375. The cells were treated with extracts in 1:3 serial dilutions with maximum concentrations of 5 mg/mL for hot water and GI extracts or 2 mg/mL for ethanol and methanol extracts. The data shown are the absolute absorbance reading of the plates.

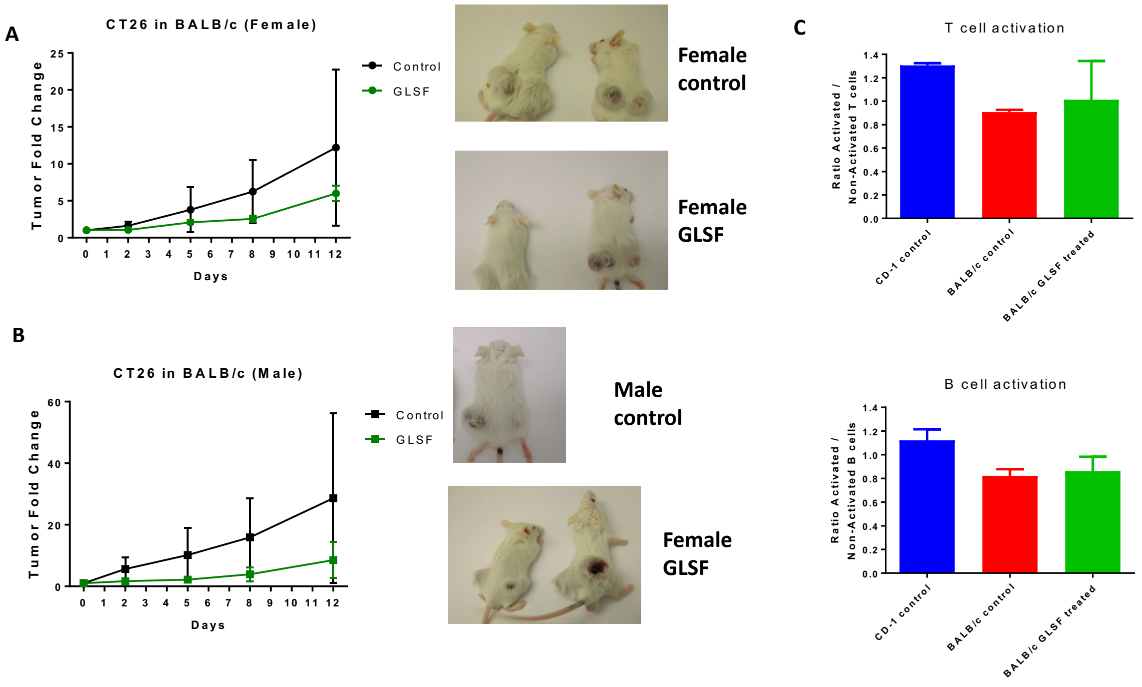

Supplementary Fig. S3 Pilot efficacy study of GLSF. The effects of GLSF in colon cancer syngeneic model in BALB/c mice carrying CT26 cells. Depicted are tumor growth fold changes normalized by the tumor volume measured at the day 0 for individual tumor. Mice pictures at the end of experiments are shown. (A) female data. (B) Male data. Male control n =1; Male GLSF n = 2; female control and GLSF (n =2). Two tumors were implanted in each mouse. Day 0, treatment started. (C) Effect of GLSF oral administration on spleen lymphocyte proliferation induced by Con A and LPS in CT26-bearing mice (females). Mice were killed and the spleens were removed. The splenic lymphocytes were plated in 96-well plates, 2×10^6 cells per well, with or without ConA (1 mg/L) or LPS (5 mg/L) and incubated for 72 hours. Cell proliferation will be quantified using MTS assay. T cell activation was induced by ConA; B cell activation was induced by LPS.

Preparation of spleen lymphocytes

Mice were sacrificed using isoflurane. Each spleen was isolated and placed in a separate 100 um cell strainer. A syringe plunger was used to break apart the spleen and the tissue was washed with DMEM complete medium. The single cell-suspension was collected in a 50 mL tube. The tissue was washed two additional times. The suspension was centrifuged at 800 g for 3 minutes at room temperature and the supernatant was discarded. The pellet was suspended in ACK lysis buffer (ThermoFisher, CAT # A10492-01) to lyse red blood cells and incubated at room temperature for 5-10 minutes. Medium was added to the cells and centrifuged at 800 g for 3 minutes at room temperature. The supernatant was discarded, and the pellet was resuspended in 3 mL of medium. Cells were counted using an automatic cell counter.

Lymphocyte proliferation assay

Spleen cells isolated from mice were seeded at a density of 2×10^6 cells per 200 uL per well in 96-well tissue culture plates. Concanavalin (Con A) (Sigma, CAT # C5275) at 1 ug/mL and Lipopolysaccharide (LPS) (Sigma, CAT #L6529 / Sigma, CAT #L6143) at 5 ug/mL was added to stimulate the activation of T

and B-cells, respectively. Cells were incubated at 37°C with 5% CO_2/95% air for 72 hours. The Promega CellTiter 96 AQueous Nonradioactive Cell Proliferation Assay Kit (MTS) assay was used to estimate cell proliferation.
